# Bacteriophage Cocktail-Mediated Inhibition of *Pseudomonas aeruginosa* Biofilm on Endotracheal Tube Surface

**DOI:** 10.3390/antibiotics10010078

**Published:** 2021-01-15

**Authors:** Viviane C. Oliveira, Ana P. Macedo, Luís D. R. Melo, Sílvio B. Santos, Paula R. S. Hermann, Cláudia H. Silva-Lovato, Helena F. O. Paranhos, Denise Andrade, Evandro Watanabe

**Affiliations:** 1 Human Exposome and Infectious Diseases Network—HEID, School of Nursing of Ribeirão Preto, University of São Paulo, Bandeirantes Avenue 3900, Ribeirão Preto, São Paulo 14040-904, Brazil; vivianecassia@usp.br (V.C.O.); paularegina@unb.br (P.R.S.H.); dandrade@eerp.usp.br (D.A.); 2 Department of Dental Materials and Prostheses, School of Dentistry of Ribeirão Preto, University of São Paulo, Café Avenue S/N, Ribeirão Preto, São Paulo 14040-904, Brazil; anapaula@forp.usp.br (A.P.M.); chl@forp.usp.br (C.H.S.-L.); helenpar@forp.usp.br (H.F.O.P.); 3 Centre of Biological Engineering—CEB, University of Minho, 4710-057 Braga, Portugal; lmelo@deb.uminho.pt (L.D.R.M.); silviosantos@deb.uminho.pt (S.B.S.); 4 Department of Nursing, University of Brasília, Distrito Federal, Brasília 72220-275, Brazil; 5 Department of Restorative Dentistry, School of Dentistry of Ribeirão Preto, University of São Paulo, Café Avenue S/N, Ribeirão Preto, São Paulo 14040-904, Brazil

**Keywords:** bacteriophage, biofilm, *Pseudomonas aeruginosa*, endotracheal tube

## Abstract

Although different strategies to control biofilm formation on endotracheal tubes have been proposed, there are scarce scientific data on applying phages for both removing and preventing *Pseudomonas aeruginosa* biofilms on the device surface. Here, the anti-biofilm capacity of five bacteriophages was evaluated by a high content screening assay. We observed that biofilms were significantly reduced after phage treatment, especially in multidrug-resistant strains. Considering the anti-biofilm screens, two phages were selected as cocktail components, and the cocktail’s ability to prevent colonization of the endotracheal tube surface was tested in a dynamic biofilm model. Phage-coated tubes were challenged with different *P. aeruginosa* strains. The biofilm growth was monitored from 24 to 168 h by colony forming unit counting, metabolic activity assessment, and biofilm morphology observation. The phage cocktail promoted differences of bacterial colonization; nonetheless, the action was strain dependent. Phage cocktail coating did not promote substantial changes in metabolic activity. Scanning electron microscopy revealed a higher concentration of biofilm cells in control, while tower-like structures could be observed on phage cocktail-coated tubes. These results demonstrate that with the development of new coating strategies, phage therapy has potential in controlling the endotracheal tube-associated biofilm.

## 1. Introduction

Ventilator-associated pneumonia (VAP) is a serious concern in critically ill patients occurring within the 48 h period following endotracheal intubation. The current COVID-19 pandemic is a predisposing factor for VAP, since it often requires mechanical ventilation, thus increasing the incidence and relevance of this infection [1]. VAP frequently involves high morbidity and excessive healthcare costs, and its incidence increases with the duration of ventilation [2,3,4]. The role of the endotracheal tube-associated biofilms in VAP etiology has been largely discussed. Commonly, biofilms on the device surface appear rapidly after intubation, promote a global covering of the internal side, and remain attached even after suctioning [2,5]. These biofilms represent a persistent source of pathogenic bacteria that can invade the lower airways, colonizing the lungs and causing VAP [6].

Strategies for biofilm growth inhibition on the endotracheal tube (ET) surface involve mainly suction systems [7], mucus shavers [8], and antimicrobial coatings [9,10,11,12]. Regarding biofilm removal, well-established strategies were not previously described in scientific literature, and direct administration of aerosolized antibiotics [13], cationic peptides [14], and ionized gas [15] have been suggested to control mature biofilm. Nonetheless, the available methods for both inhibiting and removing biofilm are not widely effective in controlling the microorganism layers on the ET surface, and innovative approaches to treat or prevent this contamination source should be investigated.

Even though the ET colonization is polymicrobial, the aerobic nosocomial bacterium *Pseudomonas aeruginosa* has been suggested to play a dominant role in the infection etiology [5,16,17]. In addition, *P. aeruginosa* has shown an enhanced ability to form huge biofilms and to develop antibiotic resistance, which in turn can be considered factors that ensure persistent infection [18,19]. Efforts have been made to reduce complications associated with *P. aeruginosa* colonization in artificial airways; nonetheless, none of them seem to be largely effective [3,20,21].

Considering the successful use of phage therapy in the treatment of *P. aeruginosa* acute respiratory infection in animal models [22], the use of phages is a promising and challenging alternative to deal with the ET-associated biofilms, mainly those formed by antibiotic-resistant strains. The advantages of using phage therapy involve low damage to the host microbiota, ability to self-replicate in the presence of host cells, host specificity, rapid selection and characterization, and low cost [23]. Aiming at treating acute infections caused by nosocomial pathogens, Aleshkin et al. reported that intragastric administration of a phage cocktail in patients with mechanical ventilation promoted an important reduction in bacterial burden [24]. Furthermore, the anti-biofilm activity of recently characterized new phages was demonstrated in vitro in an ET-associated *P. aeruginosa* biofilm model [25]. The authors demonstrated an extensive lytic activity with multidrug-resistant *P. aeruginosa* biofilm, suggesting that the new phages might be considered as good candidates for therapeutic studies [25]. Although encouraging results have revealed the anti-biofilm effect of the phage therapy, the action of immobilized phages on the ET surface, to control biofilm development, is unclear.

Along these lines, it is essential to clarify whether phages could be used for both controlling and preventing ET-associated *P. aeruginosa* biofilm. In the present study, anti-biofilm activity of five recently characterized phages was evaluated by a high content screening assay. Subsequently, two phages were selected as cocktail components and applied as a preventive strategy to inhibit bacteria colonization in a dynamic biofilm model simulating endotracheal intubation. The null hypothesis of this study was that there is no difference in *P. aeruginosa* biofilm when challenged with bacteriophages.

## 2. Results

### 2.1. Screening Phages for Anti-Biofilm Activity

**Figure 1:**
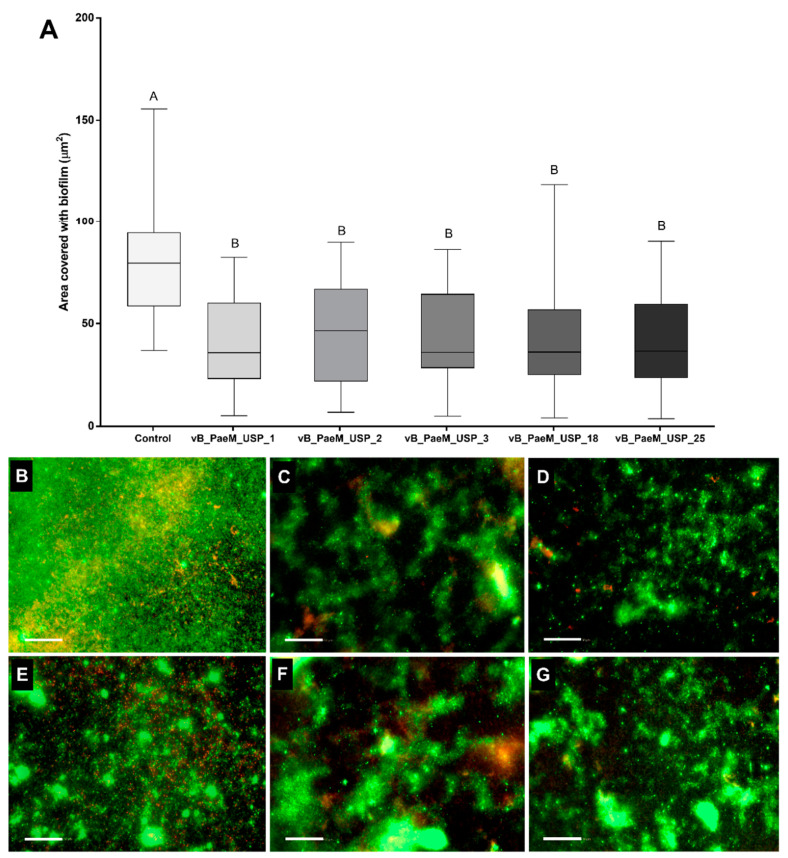
(**A**) Biofilm-covered areas, expressed in µm^2^, after phage treatment. Comparisons were conducted among groups by means of multiple comparisons considering strains and bacteriophages in a generalized linear model with Bonferroni correction. ^AB^ Different capital letters indicate statistically significant differences (*p* < 0.05). (**B**–**G**) Representative fluorescent images of *P. aeruginosa* illustrate the control group (**B**) and the action of phages vB_PaeM_USP_1 (**C**), vB_PaeM_USP_2 (**D**), vB_PaeM_USP_3 (**E**), vB_PaeM_USP_18 (**F**), and vB_PaeM_USP_25 (**G**). Scale bar = 50 µm.

An initial screening was performed to select phages with stronger anti-biofilm activity. Biofilm-covered areas showed a significant reduction after phage treatment in 4/15 *P. aeruginosa* strains (Table S1), in which three were classified previously as multidrug-resistant [25]. Biofilm areas of four other *P. aeruginosa* strains were lower, but the difference was not significant. Even though phage infectivity had been previously determined, seven *P. aeruginosa* strains were not affected by the phage treatment. The analysis of the biofilm-covered areas indicated a statistically significant difference (*p* < 0.05) between phage-treated and control; however, statistically significant differences were not observed among the five different phages (Figure 1A). Therefore, based on the broader lytic spectrum of the phages with multidrug-resistant strains; the efficiency of plating and genomic differences, reported by Oliveira et al. [25]; and the anti-biofilm activity presented here (Figure 1B–G), the phages vB_PaeM_USP_2 and vB_PaeM_USP_18 were selected to compose a cocktail in the assays involving dynamic biofilm growth on the ET surface.

### 2.2. Replication of ET Adsorbed Phage During Biofilm Growth

**Figure 2:**
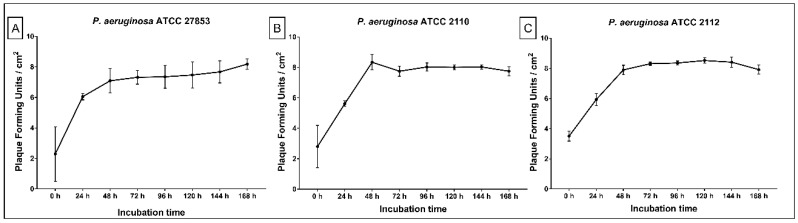
Phage cocktail presence on tube surfaces over 24 to 168 h of dynamic biofilm growth. (**A**) *P. aeruginosa* ATCC 27853; (**B**) *P. aeruginosa* ATCC 2110; (**C**) *P. aeruginosa* ATCC 2112.

The phage cocktail that was adsorbed to the ET clearly showed the ability to replicate, since the number of phage particles increased over time. The initial log 3 phage population (0 h) was able to replicate in the presence of the biofilm cells, increasing to log 6 at 24 h and log 8 at 48 h. After 48 up to 168 h, phage concentration remained almost constant without variations among the strains (Figure 2A–C).

### 2.3. Phage Cocktail Effect on P. aeruginosa Biofilms

**Figure 3:**
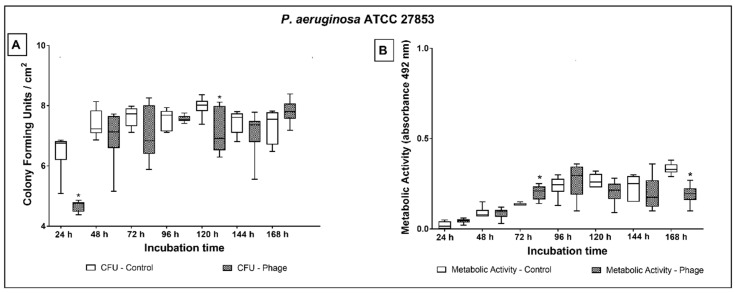
Biofilm growth (**A**) and metabolic activity (**B**) of *P. aeruginosa* ATCC 27853 over 24 to 168 h of dynamic biofilm growth on non-coated and phage cocktail-coated tubes. Comparisons were conducted among groups, at each time point, by means of multiple comparisons considering strains and phage cocktail treatment in a generalized linear model with Bonferroni correction. * indicates statistically significant difference at each time point (*p* < 0.05).

**Figure 4:**
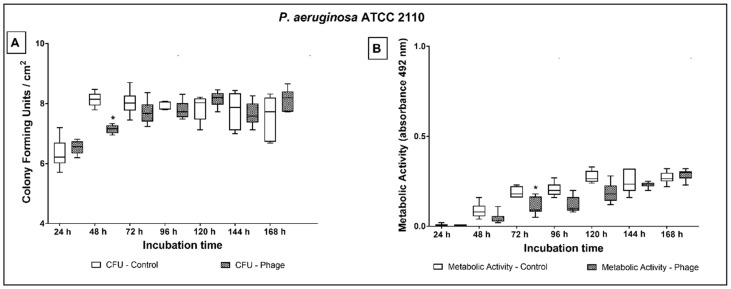
Biofilm growth (**A**) and metabolic activity (**B**) of *P. aeruginosa* ATCC 2110 over 24 to 168 h of dynamic biofilm growth on non-coated and phage cocktail-coated tubes. Comparisons were conducted among groups, at each time point, by means of multiple comparisons considering strains and phage cocktail treatment in a generalized linear model with Bonferroni correction. * indicates statistically significant difference at each time point (*p* < 0.05).

**Figure 5:**
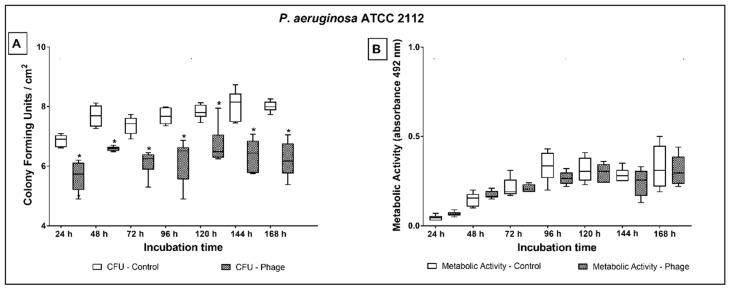
Biofilm growth (**A**) and metabolic activity (**B**) of *P. aeruginosa* ATCC 2112 over 24 to 168 h of dynamic biofilm growth on non-coated and phage cocktail-coated tubes. Comparisons were conducted among groups, at each time point, by means of multiple comparisons considering strains and phage cocktail treatment in a generalized linear model with Bonferroni correction. * indicates statistically significant difference at each time point (*p* < 0.05).

The in vivo contamination of an ET was mimicked using a continuous biofilm model system. Biofilm growth rates on non-coated tubes were similar among the three strains. However, on phage-coated tubes, a different growth pattern among the strains was observed (Table S2). This outcome indicated that the cocktail’s action was strain dependent. Regarding metabolic activity, phage cocktail coating did not promote substantial changes in the biofilm response. Generally, the absorbance values were lower at early stages and higher in late stages of cultivation time (Figure 3B, Figure 4B and Figure 5B; Table S3).

Comparing the CFU values, *P. aeruginosa* ATCC 27853 (Figure 3A) showed a significant reduction of the microbial load on phage cocktail-coated tubes at 24 (1.8 log; *p* < 0.001) and 120 h (0.9 log; *p* = 0.035) of treatment. Even though the CFU values at 48, 72, and 96 h of treatment indicated a slight biofilm reduction on phage cocktail-coated tubes (ranging from 0.1 to 0.6 log), the microbial load did not significantly differ from non-phage coated tubes.

In comparison to control, *P. aeruginosa* ATCC 27853 had higher metabolic activity on phage cocktail-coated tubes at 72 h and lower at 168 h of culture (Figure 3B).

Regarding *P. aeruginosa* ATCC 2110, significant reduction of the microbial load was observed only at 48 h (1 log; *p* = 0.004) of treatment (Figure 4A). The strain exhibited lower metabolic activity on phage cocktail-coated tubes at 72 h of culture in comparison to non-coated tubes (Figure 4B).

The reduction of *P. aeruginosa* ATCC 2112 on phage cocktail-coated tubes ranged from 1.1 to 1.8 log (Figure 5A) during the entire treatment period (*p* ≤ 0.001). Nonetheless, no difference was observed in the evaluation of the metabolic activity (Figure 5B).

**Figure 6:**
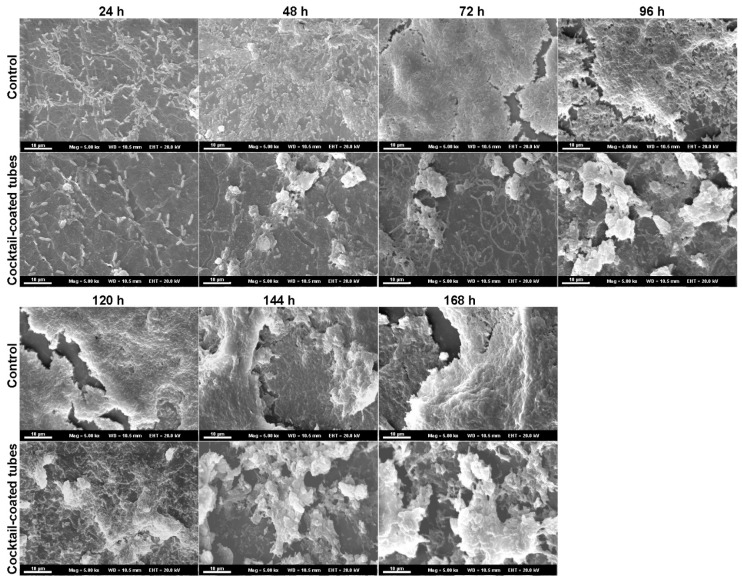
Representative scanning electron micrographs of non-coated (control) and phage cocktail-coated tubes at 24, 48, 72, 96, 120, 144, and 168 h of dynamic biofilm growth. Scale bar = 10 µm.

SEM representative biofilm images of *P. aeruginosa* ATCC 2112 for all the cultivation times are shown in Figure 6. The microscopy images of phage cocktail-coated tubes were morphologically distinct from non-phage coated ones. A higher concentration of biofilm cells was noticed covering the tube surface in the control, while tower-like structures could be observed on phage cocktail-coated tubes. In the control tubes, the biofilm grew like a homogeneous layer, while on coated tubes the highest number of cells was observed in the clusters. In addition, on control tubes an extracellular polymeric matrix covered the entire biofilm layer. On phage cocktail-coated tubes, the extracellular polymeric matrix was detected as merely covering the tower-like structures, while in the surrounding areas less matrix and fewer isolated bacteria could be observed during the entire cultivation time.

## 3. Discussion

In the current study, we assessed whether a phage treatment could efficiently reduce ET-associated biofilm. Firstly, the action of five different phages in a mature biofilm was evaluated. Subsequently, considering the lytic spectrum with multidrug-resistant strains, anti-biofilm screenings, and different efficiency of plating (EOP), two phages were selected as cocktail components and were applied as a strategy to prevent bacterial colonization and biofilm formation. Based on the results, the null hypothesis was rejected, since there were statistical differences for *P. aeruginosa* ET-associated biofilms.

According to biofilm-covered areas, our results demonstrated that phages applied solely exhibited effective anti-biofilm activity against a variety of *P. aeruginosa* strains. Nonetheless, biofilm-covered areas of seven *P. aeruginosa* strains remained unaffected when challenged with phages. Such an observation is in line with another in vitro study that showed varying degrees of biofilm disruption after phage treatment [22] and may be explained by the following reasons: (I) The efficiency of plating of the evaluated phages on five of these strains was considered low according to previously reported data [25]; (II) The antiviral mechanisms developed by the bacteria against phage adsorption, infection, and replication. The bacterial resistance systems have been extensively discussed in the scientific literature [26,27]; (III) Both treatment period and phage dosing might have been insufficient. According to Abedon, elimination of biofilms using phage therapy can require long treatment periods as well as repeated dosing [28]. Here, a single dose and treatment time was evaluated; and (IV) Staining methods and imaging tools have been considered useful for quantitative assessment and spatial structure visualization of biofilm; however, they can also result in misinterpretation of data due to laser penetration, absorption of the dye into the biomass, and auto-fluorescence [29,30]. LIVE/DEAD staining comprises two types of fluorescent stains, which differ in ability to penetrate viable and non-viable bacterial cells [31]. Here, the biofilm-covered areas were calculated according to total image fluorescence. It is known that cell concentration in biofilms can be distributed differently according to their thickness. Our image series may not have precisely recorded the biofilm density, which could in part explain unapparent anti-biofilm effects. Since agar plate counts detect all cultivable cells, the conflicting phage anti-biofilm results, reported by Oliveira et al., could be explained by the method used. The authors demonstrated superior phage anti-biofilm activity, against the same strains, by using colony forming unit counts [25]. Therefore, we consider that additional quantitative methods involving the determination of the number of viable cells by agar plate counts, flow-based cell counting, and assessment of biofilm dry mass or total protein content could lead to efficient determination of the biofilm density.

After describing the phage isolated action, a cocktail composed of vB_PaeM_USP_2 and vB_PaeM_USP_18 was investigated as an additional strategy for biofilm control on the ET surface. We hypothesized that surface coating using multiple phage strains prevents bacterial colonization considering that these two phages have different EOP, distinct lytic spectra with multidrug-resistant strains, and considerable genetic differences that could potentially lead them to bind to different receptors [32].

Efforts have been made to propose methods for phage coating in medical devices [33,34,35]. For indwelling urological devices, the phage-coating is usually obtained by physical adsorption [33,36] and hydrogel conjugation [34,37,38]. The ETs used in this study were manufactured from reinforced polyvinyl chloride (PVC), and the scientific literature describes neither phage immobilization on PVC surfaces nor on other devices designed for mechanical ventilation. In this sense, we allowed physical adsorption of 1 × 10^7^ PFU/cm^2^ to create an antimicrobial surface. After 24 h, the phage immobilized on the tube surface was 1 × 10^3^ PFU/cm^2^. The physical adsorption did not promote a large phage immobilization on the ET surface. We consider that the limited anti-biofilm effect, observed in the cocktail-coated tubes, was possibly due to the reduced phage attachment. It might be expected that by maximizing the density of the phages on the ET surface, an enhanced capacity to control *P. aeruginosa* growth would be reached. Even though the physical adsorption comprises a simple and cost-effective method for phage immobilization, the low coverage seems to be unsuitable for producing a largely anti-biofilm effect. In this sense, different studies have been proposed aiming at the development of functionalized surfaces to immobilize phages efficiently. For instance, Wang, Sauvageau, and Elias exhibited that on the plasma-treated polyhydroxyalkanoate surface, the immobilization of phage T4 was greater than on the non-treated surface [39]. Therefore, investigation of different technologies for attaching phages to PVC surfaces, as well as phages displaying plastic-binding peptides, should be performed to ensure a high phage concentration on the ET surface.

Although an initial high phage titer was not immobilized on the tube surface, in the presence of bacterial strains an increasing concentration was observed after 48 h of treatment, which confirmed the phages’ ability to replicate and compensate for the initial low dose [40]. Additionally, the 1 × 10^3^ PFU/cm^2^ was able to produce differences of bacterial colonization. According to mean differences at each specific time point, *P. aeruginosa* ATCC 27853 and *P. aeruginosa* ATCC 2112 showed evident reduction of biofilm growth on phage cocktail-coated tubes in the early stages of biofilm formation. This result can be related to the biofilm formation stage and the amount of extracellular exopolysaccharide matrix. The scientific literature has shown that inefficiency in phage penetration in mature biofilms is an important factor affecting the tolerance to phages [28]. The initial reduction in biofilm growth could be correlated both to the exponential increase of phage titer and thinner extracellular matrix layer. After 48 h, the constant titer of phages supports the idea that the thicker extracellular matrix could have hindered phage adsorption. On the other hand, a similar pattern was not observed for *P. aeruginosa* ATCC 2112, which exhibited a reduction of biofilm growth during the entire cultivation time. This distinct response can be associated with the metabolic activity of *P. aeruginosa* ATCC 2112. In comparison to *P. aeruginosa* ATCC 27853 and *P. aeruginosa* ATCC 2110, XTT assay revealed high absorbance values for *P. aeruginosa* ATCC 2112. As phages require metabolically active hosts to replicate [41], the cocktail could have more effectively infected this strain. In general, the lowest metabolic activity was observed for *P. aeruginosa* ATCC 2110, which exhibited reduction in biofilm growth only at 48 h. Taken together, these findings suggest that *P. aeruginosa* ATCC 2110 exhibits antiviral mechanisms that result in a phage-insensitive phenotype. Blocking of phage receptors, production of competitive inhibitors, prevention of bacteriophage DNA entry, slicing of bacteriophage nucleic acids, CRISPR/cas system activation, and abortive infection mechanisms are well-known bacterial resistance systems against phage infection [26,27].

The synthetic sputum medium used promotes the formation of *P. aeruginosa* aggregates with sizes similar to those observed in human cystic fibrosis lung tissue [42]. Here, however, coated and non-coated ETs had differences regarding distribution of aggregates. We suggest two different reasons to explain the formation of *P. aeruginosa* aggregates. First, on coated tubes, the formation of large bacterial aggregates, observed in SEM images, seemed to be a protection mechanism against phage invasion as it became more evident in the mature biofilms when phages reached the highest titer. Second, we speculate that this phenomenon may be caused not by the overgrowth of bacteria in some regions but by the lysis of cells by the phage cocktail causing holes on the biofilm. Structures similar to those observed here were also reported by Henriksen et al., who classified them as a defense strategy against phage infection [43]. According to the authors, the continuous phage exposure affected the biofilm growth by stimulating the formation of a highly organized and spatially heterogeneous structure.

Our results did not provide evidence regarding the different phage infection behavior of antibiotic sensitive and resistant strains. Phages have the demonstrated ability to infect both sensitive and multidrug-resistant *P. aeruginosa*. Loc-Carrillo and Abedon pointed out that resistance mechanisms against antibiotics do not affect phage infection [23]. Our results corroborate the author’s statement and indicate that phage therapy could be applied as an auxiliary method to treat infections caused by resistant bacteria. Moreover, recombinant phage-encoded enzymes could be applied directly to the tube surface as an alternative to direct phage usage.

The relevance of the present study highlights the urgent need to investigate new therapeutic strategies to control *P. aeruginosa* biofilms on the ET surface. The intubation period through an ET for ≥ 8 days represents a risk factor for VAP occurrences [4]. Here, we demonstrated that phage therapy can reduce bacterial bioburden on the ET surface and therefore might contribute to reducing VAP episodes. Nonetheless, in view of the discrepant titers applied to both strategies in the study, biofilm treatment and biofilm prevention, we were unable to determine whether the phages would be more efficient in the treatment or prophylaxis of *P. aeruginosa* biofilms. Indeed, challenges and limitations of phage therapy are evident, and the scientific literature has reported that the therapeutic or prophylactic use of phages is dependent on the application area. For instance, in the food industry, prophylactic phage administration represents a promising sustainable solution to control pathogenic bacteria and reduce the massive use of antibiotics. In this field, phages are mainly used during food production, sanitization, and preservation [44]. For both animal and human infection treatment, the therapeutic use of phages and phage-encoded enzymes, alone or in combination with antibiotics, has aroused a growing interest in their potential use against multidrug-resistant bacteria, and different routes of administration and dosage effect have been suggested [45]. In order to reduce biofilm growth on implantable medical devices, we consider that immobilization of phages or phage-encoded products, as preventive agents, might decrease colonization more effectively than using them for biofilm removal. Thus, some issues remain and should be addressed in future studies. Experimental ventilator-associated pneumonia models and preclinical assessment would be useful to clarify if the biofilm removal/inhibition promoted by phages could prevent or reduce the severity of the VAP.

## 4. Materials and Methods

### 4.1. Bacterial Strains, Growth Conditions, and Bacteriophages

All bacterial strains used in this study are listed in Table 1. Bacteria were thawed and routinely grown in tryptic soy broth (TSB; BD Difco, Sparks, MN, USA) at 37 °C with agitation. After achieving the exponential growth phase, the culture was centrifuged (4200× *g*, 5 min) and washed twice in phosphate buffered saline (PBS), pH 7.4. The bacteria inoculum was prepared considering its optical density (OD_625 nm_) measured in a spectrophotometer (Thermo Scientific, Waltham, MA, USA).

Five bacteriophages were used in this study: vB_PaeM_USP_1, vB_PaeM_USP_2, vB_PaeM_USP_3, vB_PaeM_USP_18, and vB_PaeM_USP_25. The isolation, characterization, and assessment of the lytic spectrum of the bacteriophages was described previously by Oliveira et al. [25].

### 4.2. Screening Phages for Anti-Biofilm Activity

The anti-biofilm activity of the bacteriophages was utilized against 10 clinical isolates and five strains from the American Type Culture Collection (ATCC; Table 1). Two hundred microliters of TSB containing standardized bacteria suspension (10^7^ colony forming units per milliliter—CFU/mL) was cultured (37 °C, 75 rpm) in black 96-well plates with a flat glass bottom (Corning, New York, NY, USA). After 24 h, half of the culture medium was removed, and the biofilm was supplied with freshly prepared culture medium, then plates were incubated for another 24 h. The culture medium was then discarded, and 200 µL of sterile TSB supplemented with 10^8^ plaque forming units per milliliter (PFU/mL) of bacteriophages were added to each well (6 × 10^5^ PFU/mm^2^). Culture medium without bacteriophages was used as a control. The plates were incubated for 24 h at 37 °C and 75 rpm.

To evaluate the anti-biofilm activity of the bacteriophages, the culture medium was discarded, and the wells were rinsed with 200 µL of PBS. The biofilm was stained for 15 min, protected from light, with LIVE/DEAD™ Biofilm Viability Kit (Molecular Probes, California, CA, USA) according to the manufacturer’s protocol. Afterwards, the plates were scanned, and images were randomly collected (considering peripheral and central regions) with an Operetta CLS High-Content imaging system (PerkinElmer Waltham, MA, USA) at 40× magnification with 15 fields of view/well. The biofilm-covered areas (µm^2^) were then analyzed using Harmony High Content Imaging and Analysis Software (PerkinElmer, Version 4.8, MA, USA). The assay was conducted in triplicate.

### 4.3. Phage Cocktail Pretreatment of Endotracheal Tube Surfaces

**Figure 7:**
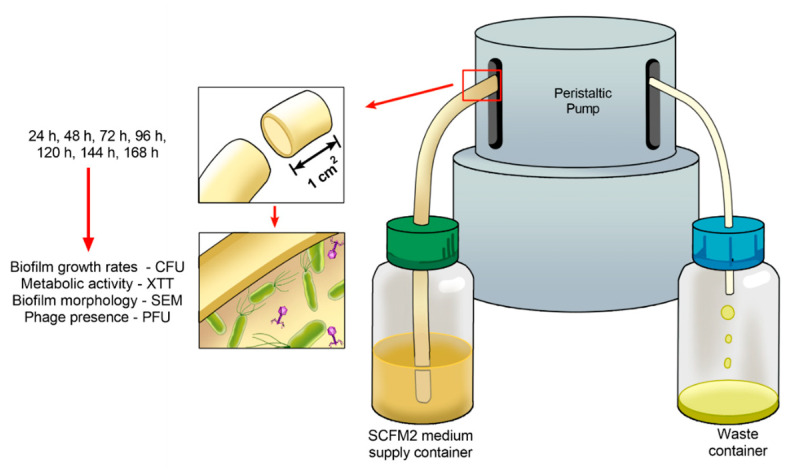
Schematic representation of the dynamic biofilm system showing that biofilm growth was monitored over 24 up to 168 h for colony forming units, metabolic activity, and biofilm morphology. Phage titer was also assessed during the entire cultivation time.

Under sterile conditions, the two ends of the ET (8.5 mm diameter, 300 mm length; Rüsh, Meridian, MS, USA) were removed to allow its connection to the tubing of the dynamic biofilm system. A phage cocktail containing 4 × 10^7^ (PFU/mL) of vB_PaeM_USP_2 and vB_PaeM_USP_18 was prepared in elution buffer (SM) (1 M Tris HCl pH 7.5, Sigma-Aldrich, Saint Louis, MO, USA; 8 mM MgSO_4_, Sigma-Aldrich; 100 mM NaCl, Dinâmica, Indaiatuba, SP, Brazil; 0.002% (*w*/*v*) gelatin, Dinâmica). Seventeen milliliters of the suspension was added to the inner part of the tube. Then, the extremities were sealed, and the device was maintained under static conditions for 24 h at room temperature. This step was employed to allow phage adsorption to the ET surface. Afterwards, the suspension was discarded, and the tube was rinsed with SM buffer in order to remove unbound phages [33]. Two fragments of 1 cm^2^ were removed from the tube to assess the presence of bacteriophages, and the flow system was mounted under sterile conditions, as demonstrated in Figure 7.

### 4.4. Developing Biofilms on Endotracheal Tube Pretreated with the Phage Cocktail

Three *P. aeruginosa* strains (ATCC 27853, ATCC 2110, and ATCC 2112) were selected for this assay. The strains ATCC 2110 (resistant to ampicillin, cefazolin, cefotaxime, cefoxitin, nitrofurantoin, trimethoprim-sulfamethoxazole, and tetracycline) and ATCC 2112 (resistant to amoxicillin-clavulanic acid, ampicillin, cefazolin, cefpodoxime, ceftriaxone, cefotaxime, cefuroxime, cefoxitin, nitrofurantoin, trimethoprim-sulfamethoxazole tetracycline, and tigecycline) were chosen due to their multidrug-resistant characteristics. The strain ATCC 27853 was selected due to the absence of antibiotic resistance. Each strain was evaluated solely in triplicate.

To simulate in vivo conditions, SCFM2 artificial sputum medium (4 g DNA salmon sperm, GoldBio, St Louis, MO, USA; 5 g swine stomach mucin, Sigma-Aldrich; 5 g casamino acids, Difco; 5.9 mg diethylenetriamine pentaacetic acid, Sigma-Aldrich; 5 g NaCl, Dynamic; 2.2 g KCl, Dynamic; 5 mL egg yolk emulsion; and 1000 mL distilled water, pH = 6.9) that mimics a cystic fibrosis model was employed [42]. After connecting to the flow system, phage cocktail-coated and non-coated tubes were supplied with a continuous flow of SCFM2 culture medium inoculated with 1 × 10^5^ CFU/mL of *P. aeruginosa* strains for 24 h. After 24 up to 168 h, the system was supplied with sterile SCFM2 culture medium without recirculation [33].

### 4.5. Analysis of the Phage Cocktail Effect on P. aeruginosa Biofilms

The phage cocktail’s ability to prevent ET colonization was determined by means of biofilm growth rates (CFU/cm^2^), metabolic activity of the biofilm (XTT), and scanning electron microscopy (SEM) at 24, 48, 72, 96, 120, 144, and 168 h under dynamic conditions. Therefore, at each time point, two fragments of 1 cm^2^ of each tube (*n* = 3) were removed for CFU counts (*n* = 6) and XTT assessment (*n* = 6). For biofilm morphology evaluation (SEM), one representative fragment was processed. The CFU and XTT methodology were performed as described previously by Oliveira et al. [25].

For CFU quantification, each fragment was transferred to a tube containing 10 mL of phosphate buffered saline (PBS). The tubes were vortexed for 60 s, sonicated (200 W, 40 kHz; Altsonic, Clean 9CA, Ribeirão Preto, SP, Brazil) for 20 min and vortexed again for 2 min to ensure detachment of all aggregated biofilm. Ten-fold dilution aliquots were seeded in tryptic soy agar (BD Difco) and incubated at 37 °C for 24 h. The number of colonies was registered and expressed as log_10_CFU/cm^2^.

For the evaluation of metabolic activity, the strains were transferred to 24-well plates containing: 948 µL PBS supplemented with 100 mM glucose (Sigma-Aldrich), 240 µL XTT 1 mg/mL (Sigma-Aldrich), and 12 µL 0.4 mM menadione (Sigma-Aldrich). The plates were incubated, protected from light at 37 °C for 2 h, and the OD_492 nm_ of the resulting solution was measured in triplicate. The mean of the readings was calculated subtracting the background absorbance.

For SEM analysis, the fragments were fixed with 2.5% glutaraldehyde (*v*/*v*) for 24 h and then dehydrated in a graded ethanol series (30%, 50%, 70%, 90%, and 100% (*v*/*v*)). After chemical drying using hexamethyldisilazane (Sigma-Aldrich), the specimens were mounted on an aluminum specimen holder and gold coated. The surface morphology of the biofilms was examined at a magnification of 3000× under high vacuum with a scanning electron microscope (EVO 10, CARL ZEISS, Jena, Germany).

### 4.6. Replication of ET Adsorbed Phage During Biofilm Growth

The replication (infection ability) of phages from the cocktail that were adsorbed on the ET surface was confirmed, at all the time points (from 0 to 168 h), by double-layer-agar plating (tryptic soy agar soft (0.8% agar)—TSAS; BD Difco) [46]. In brief, the suspension employed for CFU quantification was centrifuged and diluted in SM buffer (10^0^–10^−6^). Ten microliters were dropped onto a TSAS medium, with *P. aeruginosa* lawns, and incubated at 37 °C for 24 h. After the incubation period, the phage titer (PFU/mL) was determined by the number of phage plaques observable on the TSAS.

### 4.7. Statistical Analysis

The adherence of the data to normal distribution (Shapiro–Wilk test) and homogeneous variance (Levene test) was tested. The data set did not exhibit normal distribution and were analyzed by multiple comparisons considering strains and bacteriophages, at specific time points, in a generalized linear model with Bonferroni correction. Comparisons among time points were not conducted in view of significantly phenotypic changes in biofilm growth. The statistical tests were performed through the IBM SPSS Statistics 25.0 software (IBM Corp Armonk, NY, USA). The significance level was set to 0.05.

## 5. Conclusions

This study is the first step toward enhancing our understanding of biofilm growth in phage-coated ETs. The observed reduction depicts a favorable result but is not enough, suggesting that phages may be used not as an alternative but as a complementary strategy to control biofilms on ET, which can be improved with a better immobilization method. Since this low number of adherent phages caused significant changes in treatment, even better results are expected with an increased immobilization method. Furthermore, special attention should be paid to the potential development of phage resistance mechanisms, since over time phage treatment favors phage-insensitive phenotype development.

## Figures and Tables

**Table 1:** Bacterial strains used in the study.

Isolates	Source	Antibiotic Resistance *	Reference
*P. aeruginosa*_Mi_1 ^†^	Blood	S	[25]
*P. aeruginosa*_Mi_2 ^†^	Sputum	S	[25]
*P. aeruginosa*_Mi_6 ^†^	Urine	S	[25]
*P. aeruginosa*_Mi_7 ^†^	Sputum	S	[25]
*P. aeruginosa*_Ba_164 ^†^	Prosthetic biofilm	S	[25]
*P. aeruginosa*_Ba_168 ^†^	Prosthetic biofilm	S	[25]
*P. aeruginosa*_Ba_169 ^†^	Prosthetic biofilm	S	[25]
*P. aeruginosa*_Trac_20 ^†^	Tracheal secretion	S	[25]
*P. aeruginosa*_Trac_23 ^†^	Tracheal secretion	S	[25]
*P. aeruginosa*_Ren_1 ^†^	Saliva	S	[25]
*P. aeruginosa*_ATCC 27853 ^‡^	Blood	S	[25]
*P. aeruginosa*_ATCC 2108 ^‡^	Sputum	AMK, CFZ, CTX, GEN, IMP, TGC	[25]
*P. aeruginosa*_ATCC 2110 ^‡^	Sputum	AMP, CFZ, CTX, FOX, NIT, TGC, SXT	[25]
*P. aeruginosa*_ATCC 2112 ^‡^	Sputum	AMC, AMP, CFZ, CPD, CRO, CTX, CXM, FOX, NIT, SXT, TET, TGC,	[25]
*P. aeruginosa*_ATCC 2113 ^‡^	Sputum	AMP, AMC, CFZ, CTX, NIT, SAM, SXT	[25]

^†^ Human Exposome and Infectious Diseases Network collection. ^‡^ American Type Culture Collection. * S: Susceptible to all antimicrobial agents tested; AMC: amoxicillin-clavulanic acid; AMK: amikacin; AMP: ampicillin; CFZ: cefazolin; CPD: cefpodoxime; CRO: ceftriaxone; CTX: cefotaxime; CXM: cefuroxime; FOX: cefoxitin; GEN: gentamicin; IMP: imipenem; NIT: nitrofurantoin; SAM: ampicillin-sulbactam; SXT: trimethoprim-sulfamethoxazole; TET: tetracycline; TGC: tigecycline.

## Supplementary Materials

The following are available online at https://www.mdpi.com/2079-6382/10/1/78/s1, Table S1: Biofilm-covered areas (µm^2^) of *Pseudomonas aeruginosa* strains after 24 h in the presence of five different bacteriophages. Table S2: Colony forming units (log_10_CFU/cm^2^) of different *Pseudomonas aeruginosa* strains after 24, 48, 72, 96, 120, 144, and 168 h of culture in continuous flow, on the surface of endotracheal tubes, in the presence and absence of bacteriophage cocktail. Table S3: Metabolic activity (absorbance at 492 nm) of different *Pseudomonas aeruginosa* strains after 24, 48, 72, 96, 120, 144, and 168 h of culture in continuous flow, on the surface of endotracheal tubes, in the presence and absence of bacteriophage cocktail.
